# Ecology of an ocelot population at the northern edge of the species’ distribution in northern Sonora, Mexico

**DOI:** 10.7717/peerj.8414

**Published:** 2020-01-20

**Authors:** James C. Rorabaugh, Jan Schipper, Sergio Avila-Villegas, Jessica A. Lamberton-Moreno, Timothy Flood

**Affiliations:** 1 Saint David, AZ, USA; 2 Arizona Center for Nature Conservation, Phoenix Zoo, South Mountain Environmental Education Center, Phoenix, AZ, United States of America; 3 Sierra Club, Tucson, AZ, USA; 4 Coalition for Sonoran Desert Protection, Tucson, AZ, USA; 5 Friends of Arizona Rivers, Phoenix, AZ, USA

**Keywords:** Ocelot, *Leopardus pardalis*, Ecology, Habitat, Sonora, Behavior, Conservation

## Abstract

We used data from eight years of camera trapping at Rancho El Aribabi, a cattle ranch and conservation property in northern Sonora, Mexico, to examine the ecology of the northern-most known breeding population of ocelots (*Leopardus pardalis*). Ocelots were found mostly in two discrete and disjunct areas: a riverine riparian canyon at just less than 1,000 masl elevation and along arroyos in an oak-mesquite savanna in the Sierra Azul at 1,266–1,406 masl. An ocelot was also detected at a site between those two areas, in an area of a Sonoran desertscrub-foothills thornscrub ecotone at 1,300 masl. At least 18 ocelots, both males and females, were detected during the 2007–2011 and 2014–2018 sampling periods. A female with a kitten was documented in 2011. No individual ocelots were photographed in both areas, which are separated by a minimum of 11.29 km, and no individuals were photographed in both time periods. In a binary logistic regression, key environmental variables predicting ocelot presence were, in order of importance, distance to a paved road, distance to human habitation, proximity to water, and an anthropogenic influences index that was dominated by cattle. Another analysis corroborated the finding regarding ocelot presence and cattle. Contrary to previous studies, ocelot presence was not tied to vegetation cover close to the ground. We present information about the types of habitats and sites ocelots used, short-term movements, daily and seasonal activity patterns, and behavior, including occurrence of different individuals at or near the same site over short periods of time. We discuss ocelot home range, density, and movements, but small sample sizes and study design problems limit the value of estimates derived from our work. Rancho El Aribabi is a private, conservation ranch for which the owners have made voluntary conservation commitments that provide habitat and protection for ocelots and other animals and plants. This northern-most known breeding population is a likely source of ocelots that are periodically detected in southeastern Arizona. Our results should help facilitate conservation of the ocelot in other semi-arid areas of northwestern Mexico and adjacent USA.

## Introduction

The ocelot (*Leopardus pardalis*) is a medium-sized spotted cat (most adults weigh 8–10 kg) distributed from southern Texas and southern Arizona south to northern Argentina and the Caribbean islands of Margarita and Trinidad (Sunquist & Sunquist, 2002; Culver, 2016). Within that distribution, ocelots use a wide variety of habitats, ranging from wet tropical forests to arid thornscrub, as well as oak, pine, and fir montane forests, riparian corridors, and coastal grasslands, marshes, and mangrove swamps from sea level to over 3,000 masl elevation (López-González, Brown & Gallo-Reynoso, 2003; Avila-Villegas & Lamberton-Moreno, 2013; Caso, 2013; Aranda et al., 2014; Paviolo et al., 2015; Culver, 2016). In the northern portions of its distribution, the ocelot follows both the eastern and western flanks of Mexico into the United States (USA), largely avoiding the Sierra Madre Occidental and Oriental and the high Mexican Plateau (Sunquist & Sunquist, 2002). As of 2015, a breeding population of 53 individuals existed in southern Texas (26–27°N; US Fish and Wildlife Service, 2016). The northern-most breeding ocelots were documented by Avila-Villegas & Lamberton-Moreno (2013) at Rancho El Aribabi, Sonora, Mexico (30.8°N), about 68 km SE of the USA-Mexico border at Nogales, Sonora. The second most northerly evidence of breeding ocelots was a female and a kitten photographed on Rancho Pueblo Viejo in the lower Río Bavispe Valley, Sonora (29.6°N; T Van Devender, 2019, unpublished data).

Of 17 ocelots reported from Arizona from 1887 to 2019, sex was determined for 12; 11 were males and a lactating female was present in the San Pedro River Valley, roughly 95 km NE of Rancho El Aribabi, circa 1980–1985 (Culver, 2016; E Fernandez, 2019, pers. comm.). However, four of those ocelots reported from the 1980–1985 period, including the lactating female, were based on skins and the recollections of trappers. Localities may have been where the trappers resided rather than the collection sites (D Brown, pers. comm., 2018). At the Northern Jaguar Reserve, Sonora, about 215 km SE of Rancho El Aribabi, Gómez-Ramírez, Gutiérrez-González & López-González (2017) reported an apparently thriving population of ocelots inhabiting foothills thornscrub and oak woodlands. T Van Devender (2019, unpublished camera trap data) showed that ocelots are widespread in eastern Sonora and occupy mountains and river valleys vegetated with tropical deciduous forest, foothills thornscrub, oak woodland, and pine-oak forest, as well as riparian corridors that traverse those vegetation communities.

The ocelot is listed in the USA and Mexico as an endangered species (US Fish and Wildlife Service, 2016; SEMARNAT, 2010). The Convention on International Trade in Endangered Species of Wild Fauna and Flora (CITES, 2005) recognizes it as a species threatened with extinction and as an appendix 1 species for which trade is restricted. The ocelot is listed as a species of Least Concern on the IUCN Red List of Threatened Species (Paviolo et al., 2015).

**Figure 1:**
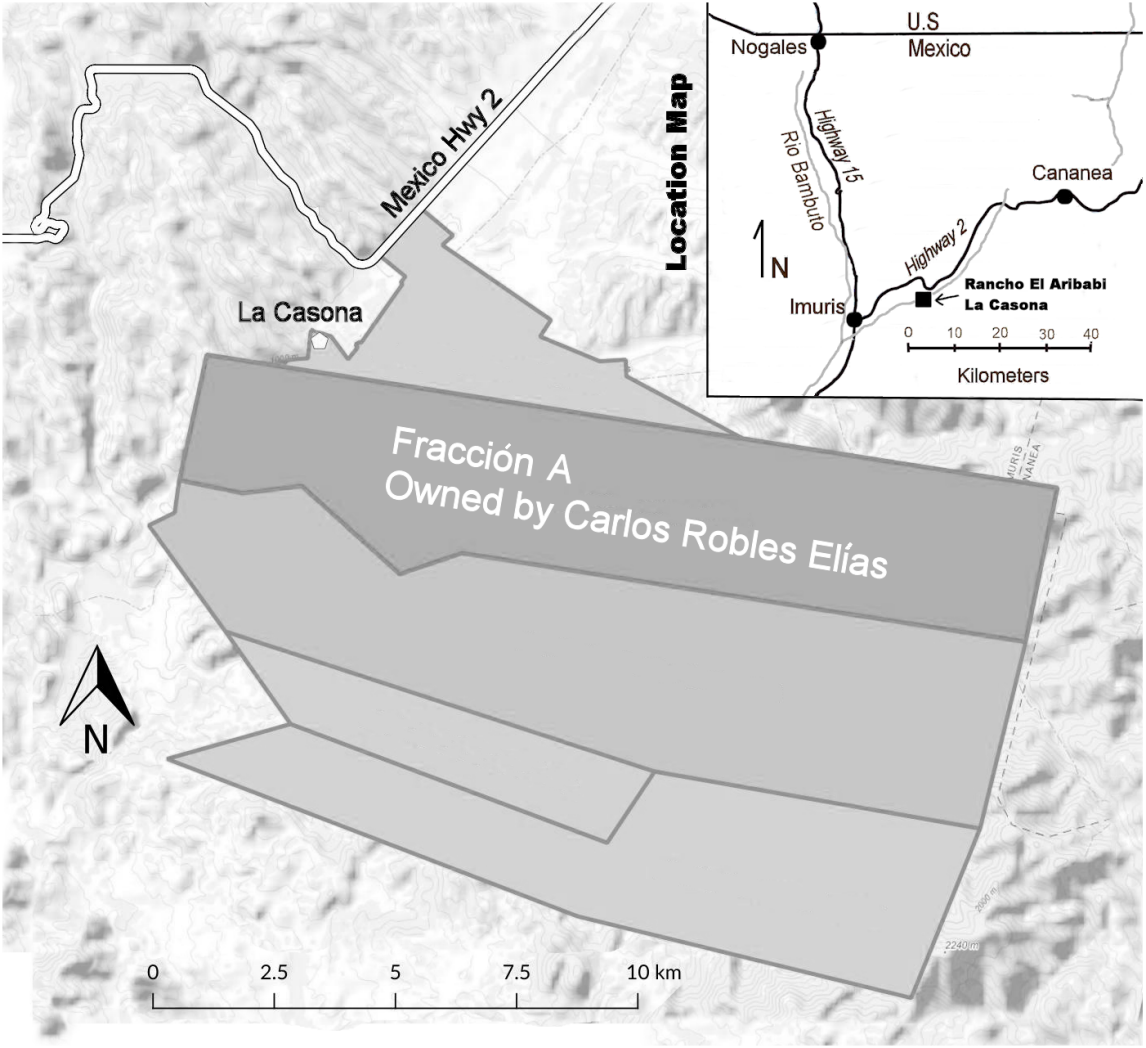
Rancho El Aribabi location map, including fraccion A (4,046 ha) where we conducted most of our work.

The purpose of our study was to explore the ecology of the northern-most known breeding population of ocelots at Rancho El Aribabi, Sonora (Fig. 1). Previous reports of ocelots in the area include the work of Avila-Villegas & Lamberton-Moreno (2013) and hunters who reportedly took ocelots in the Sierra Azul in 1966 and 1974 (López-González, Brown & Gallo-Reynoso, 2003). Rancho El Aribabi includes a portion of the Sierra Azul. Grigione et al. (2009) and Stoner et al. (2014) identified the Ímuris to Cananea area, which includes our study area, as an important habitat corridor for jaguars (*Panthera onca*) and ocelots, connecting habitat and populations to the south with the current northern extent of the distributions of these species in the USA. We believe the ocelot population at Rancho El Aribabi is a likely source of ocelots that move into southeastern Arizona, and a better understanding of ocelot habitat use, movements, activity patterns, and other ecological attributes at Rancho El Aribabi will facilitate management and conservation of this endangered spotted cat in both northwestern Mexico and southeastern Arizona.

## Materials & Methods

### Study area

**Figure 2:**
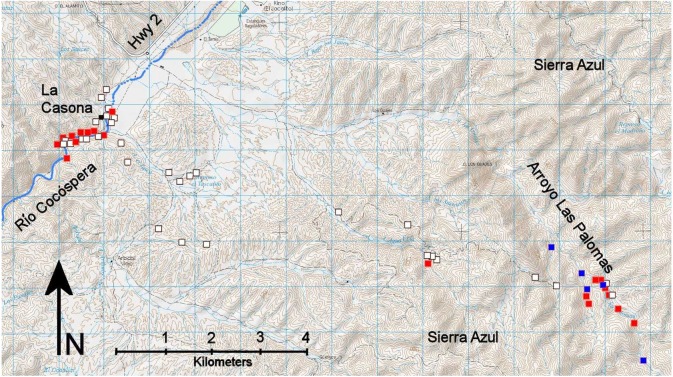
The Rancho El Aribabi study area showing all 55 camera sites and 24 ocelot localities. Red squares, ocelots photographed 2015–2018; blue squares, ocelots photographed 2007–2011; white squares, no ocelots. La Casona ranch headquarters marked with a black square. Adapted from INEGI 1:50,000 topographic maps H12B51 and H12B52.

Rancho El Aribabi is a 15,700-ha group of cattle-ranching properties situated in the Sky Island region of northern Sonora, Mexico (Fig. 1). Our study area is shown in Fig. 2, which encompasses 4,046 ha of the ranch that is owned and managed by Carlos Robles Elías. “La Casona”, the ranch headquarters, is located 59 km SSE of the USA-Mexico border at Nogales, Sonora-Arizona. The study area is bordered by the western slopes of the Sierra Azul and the eastern and southern slopes of the Sierra Los Pinitos. The western edge of the study area includes a portion of the Río Cocóspera, with a perennial reach that begins in a large ciénega (spring-fed wetland) adjacent to La Casona and runs through a river canyon between the Sierras Azul, Los Pinitos, and de la Madera. Elevations in the study area extend from about 960 to 2,000 masl and the terrain is largely mountainous or rolling bajadas (alluvial fans at the base of mountains). Biotic communities range from a Sonoran desertscrub-foothills thornscrub ecotone at the very lowest elevations and on south-facing slopes, upslope through mesquite grasslands or semi-desert grasslands, Madrean oak woodlands and savanna, and pine-oak forests at the highest elevations in the Sierra Azul (Brown & Lowe, 1994). Although the vegetation communities of the study area have not been formally mapped, we estimate that approximately 50, 30, and 17 percent of the land area is vegetated in oak woodlands and savanna (with small amounts of pine-oak woodland), semi-desert or mesquite grasslands, and Sonoran desertscrub-foothills thornscrub ecotone, respectively. The small percent remaining is riparian woodland, primarily along the Río Cocóspera, composed mainly of Fremont cottonwood (*Populus fremontii*), Goodding willow (*Salix gooddingii*), and velvet mesquite (*Prosopis velutina*).

The primary land use in the study area and in this region of Sonora is cattle ranching. A system of cattle tanks, pipelines, and troughs have been established to provide water for livestock; pasture fences are maintained to manage grazing, and unpaved roads provide access to much of the property. Carlos Robles Elías primarily leases his rangeland for grazing by steers, which typically spend about a year on the ranch before being rounded up and sold. The owners, the Robles family, have devoted numerous efforts to promote conservation of the ranch’s ecosystems. In 2011, sections (hereafter fracciones) A and B of Rancho El Aribabi were designated a Protected Natural Area by the federal Mexican agency, CONANP (La Comisión Nacional de Áreas Naturales Protegidas). This is the highest designation of environmental protection conferred by the Mexican government on private lands. Fraccion A (Fig. 1), which is owned and managed by Carlos Robles Elías, includes the Río Cocóspera canyon and extends eastward into the Sierra Azul to include Arroyo Las Palomas and much of its watershed, an area where ocelots and jaguars were documented by Avila-Villegas & Lamberton-Moreno (2013) during a camera-trap project from 2007 to 2011. The Río Cocóspera and Arroyo Las Palomas are included in a conservation zone with voluntary restrictions on human uses; limited cattle grazing and game hunting are allowed.

### Camera traps

**Figure 3:**
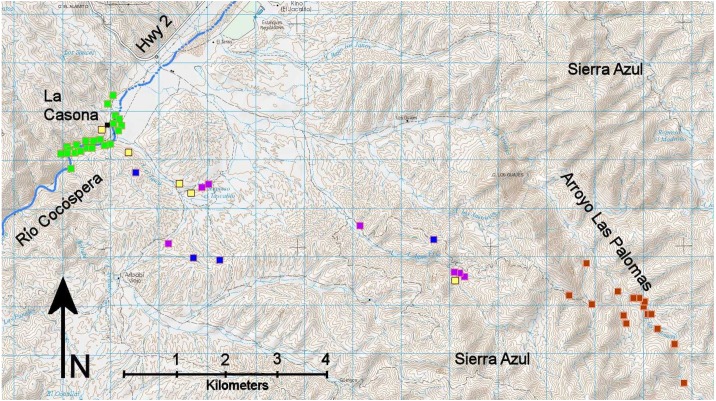
Camera sites by location type. Green, Río Cocóspera; blue, ridgeline; purple, livestock water sources; yellow, upland; rust, Arroyo Las Palomas and tributaries. La Casona ranch headquarters marked with a black square. Adapted from INEGI 1:50,000 topographic maps H12B51 and H12B52.

To record wildlife observations, we placed camera traps at 50 sites in the study area (Figs. 2 and 3, Table 1) from July 2014 to December 2018. We also report herein on ocelots recorded at five additional camera sites in the Arroyo Las Palomas area from 2007 to 2011. Cameras of a variety of manufacturers and capabilities were employed. We used primarily Stealthcam cameras with infrared night flash, but also employed Scoutguard, Cuddeback, and Moultrie white-flash cameras, as well as other infrared-flash cameras, including Bushnell, Moultrie, Reconyx, and Wildview. All cameras were set to record three images per trigger, followed by a 30-second delay before the camera could be triggered again. For short periods of time, three cameras were set on video mode. Cameras were not set in pairs, a strategy often used to photograph both sides of a spotted cat to enhance identification of individuals (Karanth, Nichols & Kumar, 2004). We used no bait, scents, or other attractants at our camera sites.

**Table 1:** Camera sites. Camera site type and numbers, site descriptions, and ocelot detections, 2014–2018.

Camera site type	#Cameras	#Camera days	#Cameras with Ocelots	#Ocelot events	#Camera days/ Ocelot event	Macro vegetation community and terrain
Río Cocóspera	22	9220	10	44	209	Riparian forest, perennial stream
Ridgeline	4	919	0	0	0	Tops of ridges in mesquite grassland
Livestock water	7	2752	0	0	0	Mesquite grassland, montane or bajadas
Upland	5	940	1	2	470	Mesquite grassland, montane or bajadas
Arroyo Las Palomas and tributaries	12	7693	8	45	171	Oak-mesquite savanna, montane, intermittent to perennial stream

We did not randomize camera locations or set them in a grid during either time period. Rather, cameras were positioned mostly on trees or large shrubs and aimed at animal trails, roads, ridgelines, log crossings of the Río Cocóspera, and water sources that we anticipated would be frequented by medium to large wild mammals. During 2014 to 2018 when an ocelot was detected, we increased our density of cameras around the detection site to maximize data on the species. For data analysis we grouped cameras by site location (riparian woodland on the Río Cocóspera, ridgeline, livestock water source (earthen cattle tank or trough), upland bajada, or Arroyo Las Palomas and its tributaries in the Sierra Azul; Table 1, Fig. 3). Cameras were checked about every three months, except cameras deep into the Sierra Azul were loaded with lithium-ion, long-lasting batteries and checked every six months because that area was difficult to access. All images obtained were reviewed and catalogued. If wildlife was photographed, we noted species, group size, behavior, time, and date. We grouped images into events, defined as a series of images of the same species with no gaps between observations of more than 15 minutes. Individual ocelots were identified, if possible, based on unique spot patterns (Trolle & Kéry, 2005; Zimmermann & Foresti, 2016). Male ocelots were distinguished from females by presence of a scrotum. Wildlife other than medium to large mammals was noted as incidentals. Anthropogenic influences (people, dogs, horseback riders, horses, cattle, burros, and vehicles) were tallied by number of events, maximum group size in an event, and time in minutes of each event. The 2007 to 2011 data were treated differently; for that period, we evaluated images only of ocelots.

### Habitat characterization

Vegetation, terrain, elevation, and straight-line distances to the nearest permanent water, unpaved road, and paved road were calculated for each of the 2014–2018 camera sites. Vegetation community at each camera site was characterized at the macro level (biotic community: riparian woodland, Sonoran desertscrub-foothills thornscrub ecotone, mesquite grassland/semi-desert grassland, or oak/mesquite savanna), and site-specific level (line-intercept transects; Bonham, 1989). Site-specific vegetation measurements included two transects that began at the focal point of the camera and extended for 10 m from that focal point along randomly-selected compass directions. Percent cover of each plant species or barren ground along the transects was measured in three vertical strata: ground cover (<0.3 m), shrub layer (0.3–1.8 m), and canopy (>1.8 m). Cover for each plant species was measured using distance of intercepts along the transect, and then summed over all species and divided by the transect length (10 m) to determine percent cover, which sometimes exceeded 100% because of overlapping canopies. For analysis, we combined the lower two strata into one (added all plant distances along the transect and divided by the transect length to determine “percent cover”) to correspond to low vegetation cover often found to be important for ocelots (Sunquist & Sunquist, 2002; Horne et al., 2009). Transects were assessed in May 2018. We did not conduct vegetation transects at the 2007–2011 camera sites due to probable changes in vegetation characteristics.

To explore the relationship between habitat variables and ocelot presence or absence, we conducted a binary logistic regression in which sample points were the 2014–2018 camera locations, the dependent variable was ocelot presence/absence (1/0), and the independent variables were distance (km) to permanent water, distance (km) to a paved road, distance (km) to an unpaved road, distance (km) to a human habitation, distance (km) to a vegetation community that included Fremont cottonwood, Goodding willow, or Arizona sycamore (*Platanus wrightii*), percent canopy cover (>1.8 m), percent cover in the shrub and ground layer (<1.8 m), and an anthropogenic influences index. The latter index was calculated separately for seven anthropogenic influences (people, dogs, horseback riders, horses, cattle, burros, and vehicles) at each camera site by adding the number of events, maximum group size of each event, and number of minutes of each event for the duration any camera was operating. Those resulting numbers were converted into an index by summing the totals for all seven anthropogenic influences and dividing by the number of camera days the subject camera was operated. Logistic regression calculations were run in XLSTAT (version March 2019).

In our analysis of data collected from November 2015 to December 2018 (dates during which ocelots were detected in the 2014–2018 study), we further explored the effects of cattle by comparing ocelot events in months with and without cattle at the eight Arroyo Las Palomas and vicinity (ALP) camera sites where ocelots were detected during that time period. Our null hypothesis was that there is no difference in ocelot events at our ALP camera sites regardless of cattle presence. We tested the null hypothesis with a two-tailed *z* statistic test. We chose the Las Palomas sites for this analysis because those cameras were in a single pasture and the grazing practice typically stocked large numbers of cattle or none at all. In contrast, along the Río Cocóspera (RC), cattle numbers fluctuated, but cattle were almost always present.

### Demographics, movements, and other behavior

A 100% minimum convex polygon (MCP; Mohr, 1947) was plotted and the area of that MCP was measured for ocelots with three or more localities. Maximum distance moved was also calculated for individual ocelots photographed at two or more localities. Density of ocelots was estimated roughly by tallying the number of individual ocelots and dividing by the area in square kilometers of the study area. To assess whether our sampling was capturing all ocelots in the study area each year, numbers of ocelot individuals detected were plotted against cumulative ocelot events for the years 2007–2010 and 2016–2018. If we were capturing most or all ocelots in the study area, then the resulting functions should plateau with increasing ocelot events. For reasons detailed in the Discussion, we did not use our data to calculate precise ocelot home ranges or short-term densities.

Movements of individual ocelots were also assessed by examining data clusters of the same ocelot in temporal space, showing movements across the landscape and the time taken for those movements. We also examined interactions among ocelots that were photographed at the same camera site on or about the same day.

## Results

The cameras captured 398,989 images, 1,033 video clips, 36,131 images of wildlife (excluding incidentals), and 10,104 wildlife events over 21,535 camera-trap days from July 2014 to December 2018. Twenty species of medium to large wild mammals were recorded (Table 2). Additional vertebrate species were photographed incidentally. The cameras captured 6,706 events of anthropogenic influences (Table 2), which were dominated by cattle. Bovine events (5,557) exceeded those of even the most commonly photographed medium to large mammal (white-tailed deer [3,870 events]) reflecting land use at Rancho El Aribabi as an active cattle ranch. However, the mode of cattle operation, at least in the Sierra Azul (steer operation), resulted in periods without cattle punctuated with periods of often heavy cattle use, providing an opportunity to compare ocelot events under those differing conditions.

**Table 2:** Medium to large wild mammals and anthropogenic influences captured on our camera traps by species and ordered by number of events.

Wildlife (events/images/#camera sites)	
White-tailed Deer (*Odocoileus virginianus*) 3841∕15811∕45	Ringtail (*Bassariscus astutus*) 150∕247∕19
Javelina (*Pecari tajacu*) 2020∕9099∕43	Hog-nosed Skunk (*Conepatus leuconotus*) 108∕267∕21
White-nosed Coati (*Nasua narica*) 781∕3522∕33	Ocelot (*Leopardus pardalis*) 91∕215∕19
Coyote (*Canis latrans*) 734∕1890∕37	Hooded Skunk (*Mephitis macroura*) 80∕228∕18
Gray Fox (*Urocyon cinereoargenteus*) 537∕1132∕39	Northern Raccoon (*Procyon lotor*) 67∕164∕16
Puma (*Puma concolor*) 446∕1270∕35	Arizona Gray Squirrel (*Sciurus arizonensis*) 47∕138∕7
Rock Squirrel (*Spermophilus variegatus*) 324∕676∕20	Hooded or Hog-nosed Skunk 43∕75∕18
Antelope Jackrabbit (*Lepus alleni*) 269∕647∕9	Sonoran Opossum (*Didelphis virginiana*) 20∕49∕8
Desert Cottontail (*Sylvilagus audubonii*) 245∕478∕15	Black Bear (*Ursus americanus*) 17∕50∕5
Bobcat (*Lynx rufus*) 198∕506∕32	American Badger (*Taxidea taxus*) 11∕24∕6
Unidentified: 185∕288∕37	Western Spotted Skunk (*Spilogale gracilis*) 3∕8∕3
Anthropogenic Influences (events/# camera sites)	
Cow (*Bos taurus*) 5557/43	Vehicle 278/12
Horseback Rider (*Eques caballus* & *Homo sapiens*) 337/35	Dog (*Canis lupus*) 178/30
Human (*Homo sapiens*) 215/35	Horse 135/15
	Wild Burro (*Eques asinus*) 4/3

From July 2007 through February 2011, 47 images and 45 ocelot events were recorded at five camera sites, all at Arroyo Las Palomas or tributaries thereof. Nine individuals were identified, including five males, one female, and three of undetermined gender. Two of 45 events yielded images of insufficient quality to identify individuals, so additional individuals may have been photographed. A female with a mostly grown kitten following behind it was photographed on 12 February 2011. We sent the images to Mitch Sternberg, who studies ocelots in Texas, and he estimated the kitten was 1–2 years of age.

A total of 215 images and 91 events of ocelot were captured at 19 camera sites from November 2015 through December 2018. Ocelots were not detected by us visually or by other means (e.g., tracking), although, during our study, a Robles family member reported seeing an ocelot crossing the dirt road between La Casona and Highway 2 in 2016 (C Robles Elías, pers. comm., 2016). We recorded nine different individual ocelots, including three males, four females, and two of unknown gender; however, 28 of the events yielded images of insufficient quality to identify individuals, so additional ocelots may have been photographed. Furthermore, mammals could not be identified to species in 185 events (Table 2), and it is possible that some of those involved ocelots. Of the individuals that could be positively identified, none from 2007–2011 were the same as those photographed during 2014–2018.

Ocelot events were usually of short duration and consisted of animals walking through the camera’s view, although one ocelot was photographed while running. Events of one minute or less accounted for 132 of 136 events. Two lasted two minutes, one was three minutes, and one lasted 14 minutes. Only one event showed more than one ocelot—the aforementioned female with a kitten. The only other animal noted in an ocelot event was a small owl (Western Screech Owl (*Megascops kennicottii*) or Whiskered Screech Owl (*Megascops trichopsis*)). Ocelots were mostly photographed travelling through arroyos (65 events), ranging from very small arroyos only a meter wide (but with no discernable animal trail) to the broad floodplain of the Río Cocóspera. Five events were recorded on rarely-used dirt roads and three were along narrow animal trails. Eighteen other events showed an ocelot crossing a log over the Río Cocóspera. Fifteen of those were female R1, two were male R2, and one could not be identified. R2 stopped to sniff at the log in two events. Many other mammals used the log to cross the river, and a puma (*Puma concolor*) was photographed scratching at the place at which the ocelot later sniffed. That log was a fallen Fremont cottonwood about 0.3 m in diameter and largely free of side branches, leaving a mostly unimpeded bridge across the river. A Goodding willow log of similar diameter also provided a crossing about 3–4 m upstream of the cottonwood, but it had many side branches and ocelots were not photographed crossing on that log.

### Habitat characterization

Ocelots were photographed primarily in two locations of the study area: (1) the Río Cocóspera and its environs at elevations of 972–995 masl along a perennial reach of the river, and (2) Arroyo Las Palomas and tributaries thereof in the Sierra Azul at elevations of 1,266–1,406 masl (Fig. 2). The Río Cocóspera camera sites were primarily in mature gallery Fremont cottonwood and Goodding willow riparian forest, although cameras on the edge of the floodplain also recorded ocelots in woodlands of velvet mesquite, netleaf hackberry (*Celtis reticulata*), and other shorter trees and shrubs. At Arroyo Las Palomas and its tributaries, the vegetation was an oak (several species of *Quercus*) and velvet mesquite savanna, but ocelots were recorded along intermittent drainages that contained scattered Fremont cottonwood, Goodding willow, and Arizona sycamore with occasional perennial pools. Two ocelot events were also recorded along a small ephemeral arroyo in a Sonoran desertscrub/foothills thornscrub ecotone at 1,300 masl, about a third of the way from Arroyo Las Palomas to the Río Cocóspera (Fig. 2). Ocelots were not photographed at artificial water sources for livestock (troughs [two camera sites], earthen cattle tanks [five camera sites]), or along ridgelines in mesquite grassland (four camera sites; Table 1).

To assess the importance of eight habitat variables (a–h, described below) on ocelot presence, a binary logistic regression run on the 2014-2018 data yielded the following equation,

\begin{eqnarray*}\text{Ocelot Presence}=1/1+\exp \nolimits [-(-7.23-[6.93\times \mathrm{a}]+[6.75\times \mathrm{b}]+[0.07\times \mathrm{c}]\nonumber\\\displaystyle \quad \quad \quad -[6.13\times \mathrm{d}]-[13.63\times \mathrm{e}]-[0.01\times \mathrm{f}]-[0.02\times \mathrm{g}]-[0.13\times \mathrm{h}])] \end{eqnarray*}

where ocelot presence ranged from 1 (present) to 0 (absent), a = distance (km) to permanent water, b = distance (km) to paved road, c = distance (km) to unpaved road, d = distance (km) to human habitation, e = distance (km) to a macro-vegetation community containing Fremont cottonwood, Goodding willow, and/or Arizona sycamore, f = percent canopy cover (>1.8 m), g = percent cover in the shrub and ground layer (≤1.8 m), and h = anthropogenic influences index. Means and ranges for habitat variables and results of statistical tests are presented in Table 3. Area under the curve was 0.924 and Table 4 lists standardized coefficients. Those coefficients indicate that distance to a paved road, distance to a human habitation, proximity to permanent water, and the anthropogenic influences index, in that order, were the most important variables in predicting ocelot presence. A training sample of the model classified 70 and 89%, respectively, of negative and positive ocelot camera sites correctly (mean = 79%). Mean camera days/ocelot event for the 19 camera sites where ocelots were detected during 2015–2018 was 125. To minimize the likelihood of false negatives (categorizing an ocelot not-present site when in fact ocelots were present), camera sites where ocelots were not detected were excluded from the logistic regression if they were not operated for at least 125 days. That qualifier resulted in 20 camera sites classified in the logistic regression as ocelot non-detection sites. Sites of human habitation were ranch houses where people lived most of the time. La Casona was one of those human habitation sites, and although we describe it as a ranch headquarters, typical ranch activities (e.g., rounding up cattle, stabling horses, storing feed) did not occur there. Rather it is a place where the Robles family and their friends live or visit.

The anthropogenic influence index used in the binary logistic regression was dominated by cattle (see Table 2). Effects of cattle on ocelots was further assessed by comparing numbers of ocelot events in months with and without cattle at the eight Arroyo Las Palomas and vicinity camera sites where ocelots were photographed during 2014–2018. The number of ocelot events during months without cattle was 40 (over 27 months) versus five when cattle were present (11 months). Assuming the likelihood of an ocelot event is equal in all months, regardless of cattle presence (null hypothesis: cattle have no effect on ocelot events, or the difference between the two ratios equals zero), the likelihood of the above ratios is very low (*p* = 0.003, two-tailed test, z statistic = 2.73, SE = 0.285), thus we reject the null hypothesis.

**Table 3:** Summary statistics for the binary logistic regression of habitat characteristics and detection or not of ocelot at camera sites.

	Habitat Variable^a^ Mean (range)							
Camera sites	a	b	c	d	e	f	g	h
Ocelot detected^b^	0.22 (0–0.7)	6.32 (1.45–11.95)	0.36 (0.01–0.69)	4.91 (0.07–10.74)	0.95 (0–1)	79.1 (0–200)	23.4(0–77)	4.41 (0–43.36)
No Ocelots^c^	0.50 (0–1.53)	4.66 (1.18–11.16)	0.15 (0–0.57)	3.40 (0.11–9.69)	0.55 (0–1)	67.8 (0–155)	37.0 (4.3–102)	3.38 (0–8.76)
Hosmer and Lemeshow goodness of fit test: *df* = 8, Ch *i*^2^ = 5.695, Pr >Ch *i*^2^ = 0.681								
Area Under the Curve (AUC) = 0.924								
−2 Log(Likelihood): *df* = 8, Chi^2^ = 25.46, Pr >Chi^2^ = 0.001								

**Notes.**

a a, distance to permanent water (km); b, distance to paved road (km); c, distance to unpaved road (km); d, distance to human habitation (km); e, presence (1) or absence (0) of cottonwood, willow, or sycamore (macro vegetation community); f, % canopy cover (>1.8 m); g, % cover in the shrub and ground layer (≤ 1.8 m); and h, anthropogenic influences index.

**Table 4:** Standardized coefficients for the logistic regression analysis.

Variable^a^	Value	Standard error	Wald Chi^2^	Pr >Chi^2^	Wald lower bound (95%)	Wald upper bound (95%)
a	−2.006	1.335	2.257	0.133	−4.623	0.611
b	14.062	8.619	2.662	0.103	−2.831	30.955
c	0.191	0.307	0.385	0.535	−0.412	0.793
d	12.873	7.957	2.617	0.106	−28.469	2.723
e	0.177	0.412	0.183	0.688	−0.632	0.985
f	−0.044	0.319	0.019	0.889	−0.669	0.580
g	−0.073	0.290	0.064	0.800	−0.642	0.496
h	−0.550	0.467	1.390	0.238	−1.465	0.364

**Notes.**

a a, distance to permanent water (km); b, distance to paved road (km); c, distance to unpaved road (km); d, distance to human habitation (km); e, presence (1) or absence (0) of cottonwood, willow, or sycamore (macro vegetation community); f, % canopy cover (>1.8 m); g, % cover in the shrub and ground layer (≤1.8 m); and h, anthropogenic influences index.

### Demographics, movements, and other behaviors

**Figure 4:**
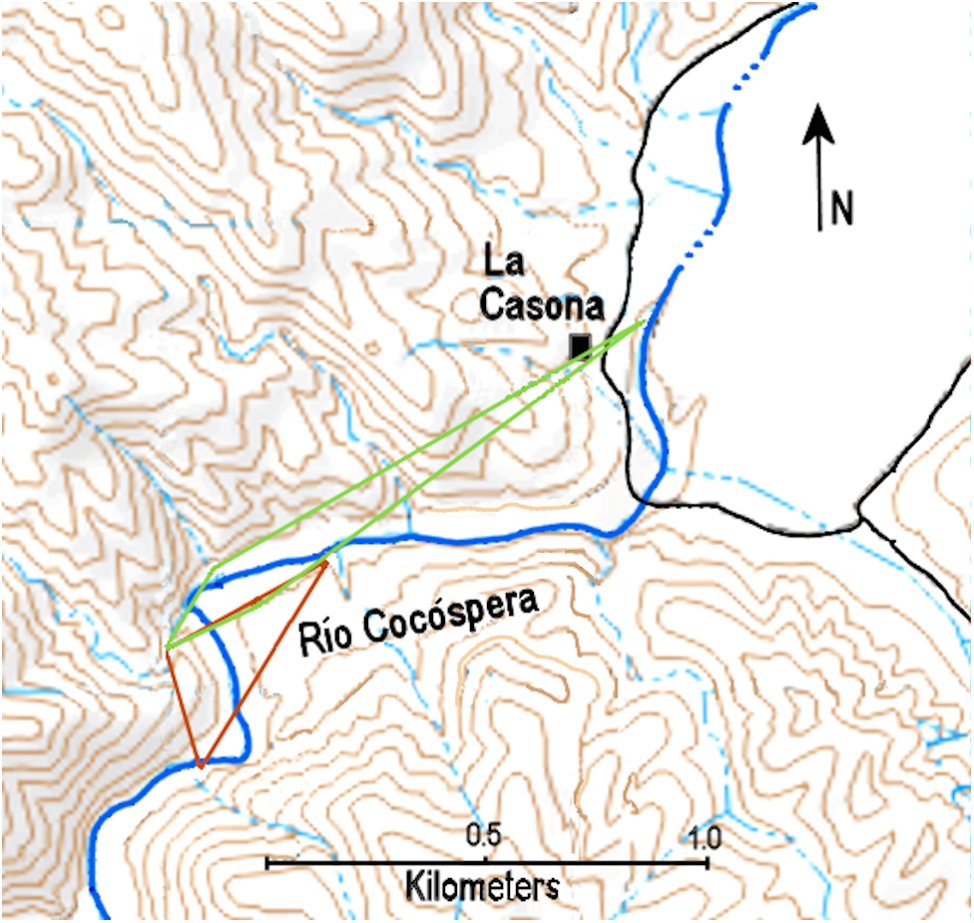
Minimum convex polygons of ocelot R1-female (brown) and R2-male (green) along the Río Cocóspera.

**Figure 5:**
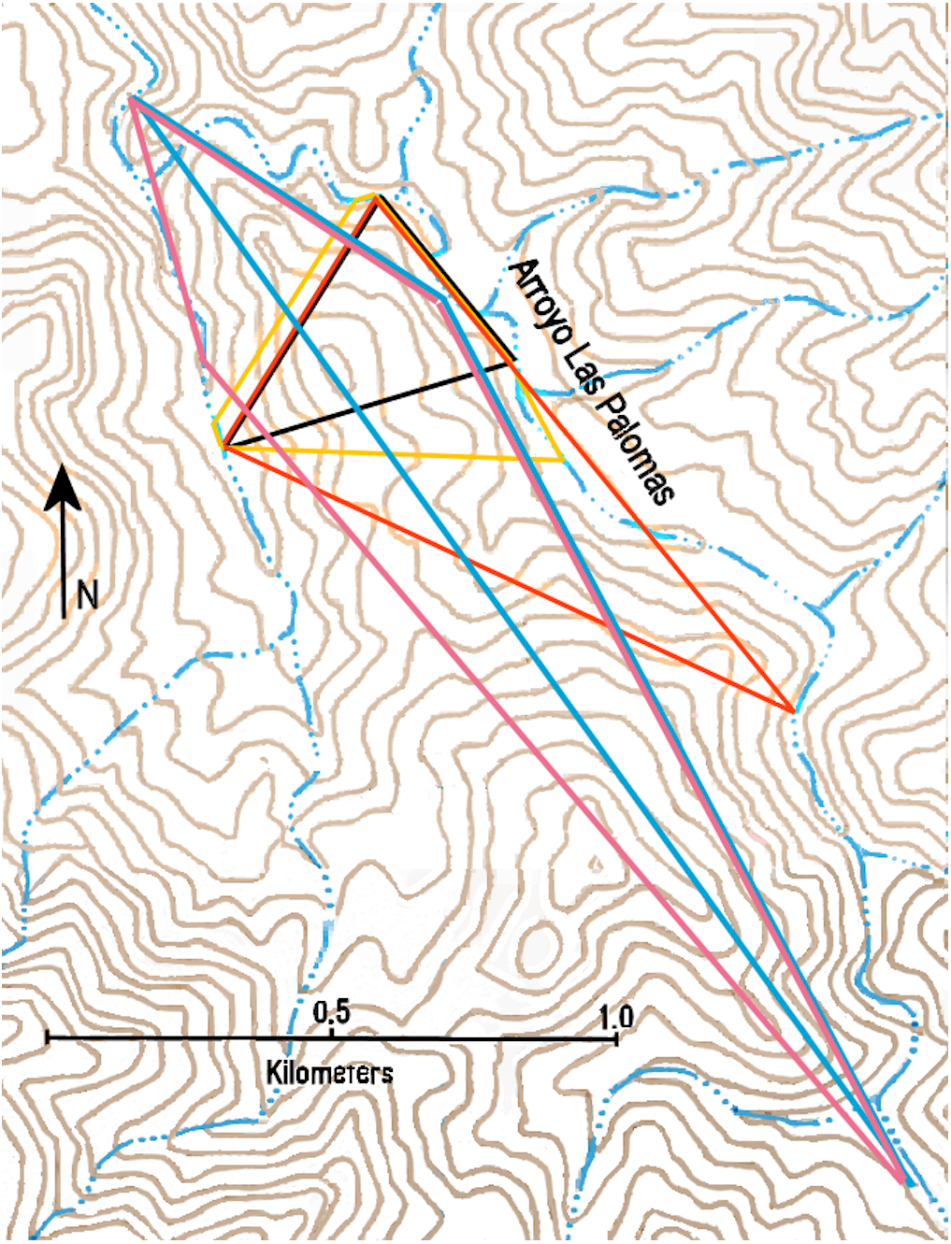
Minimum convex polygons of ocelot LP1-male (black), LP2-female (yellow), LP3-gender unknown (orange), LP6-male and LP8-female (blue), and LP7-male (pink) in the Arroyo Las Palomas area.

MCP and maximum distance moved for ten ocelots is presented in Table 5. MCPs are displayed in Figs. 4 and 5, which are based on 3–6 localities and 3–25 ocelot events, and varied from 0.06–1.19 km^2^ (mean = 0.37 km^2^) for females and 0.10–2.47 km^2^ (mean = 0.85 km^2^) for males. The mean MCP for all ten ocelots was 0.52 km^2^. Maximum distance moved for 10 ocelots detected at more than one locality during the study varied from 0.51–2.38 km (mean = 1.29 km). No ocelots were known to move between the Arroyo Las Palomas area and the Río Cocóspera camera sites, which are separated by a minimum of 11.29 km.

**Table 5:** Ocelot minimum convex polygons (MCP) and maximum distances moved. Ocelot names beginning with “R” represent animals from the Río Cocóspera; names beginning with “LP” are from Arroyo Las Palomas and its tributaries.

Ocelot	Gender	#Localities	#Events	100% MCP (km^2^)	Maximum distance moved (km)
R1	F	3	25	1.19	0.54
R2	M	5	9	2.47	1.22
LP1	M	4	6	0.10	0.55
LP2	F	6	9	0.14	0.56
LP3	?	3	4	0.28	1.16
LP4	F	3	3	0.10	1.18
LP5	F	3	5	0.06	0.51
LP6	M	3	12	0.34	2.38
LP7	M	4	17	0.51	2.38
LP8	F	3	6	0.34	2.38

**Figure 6:**
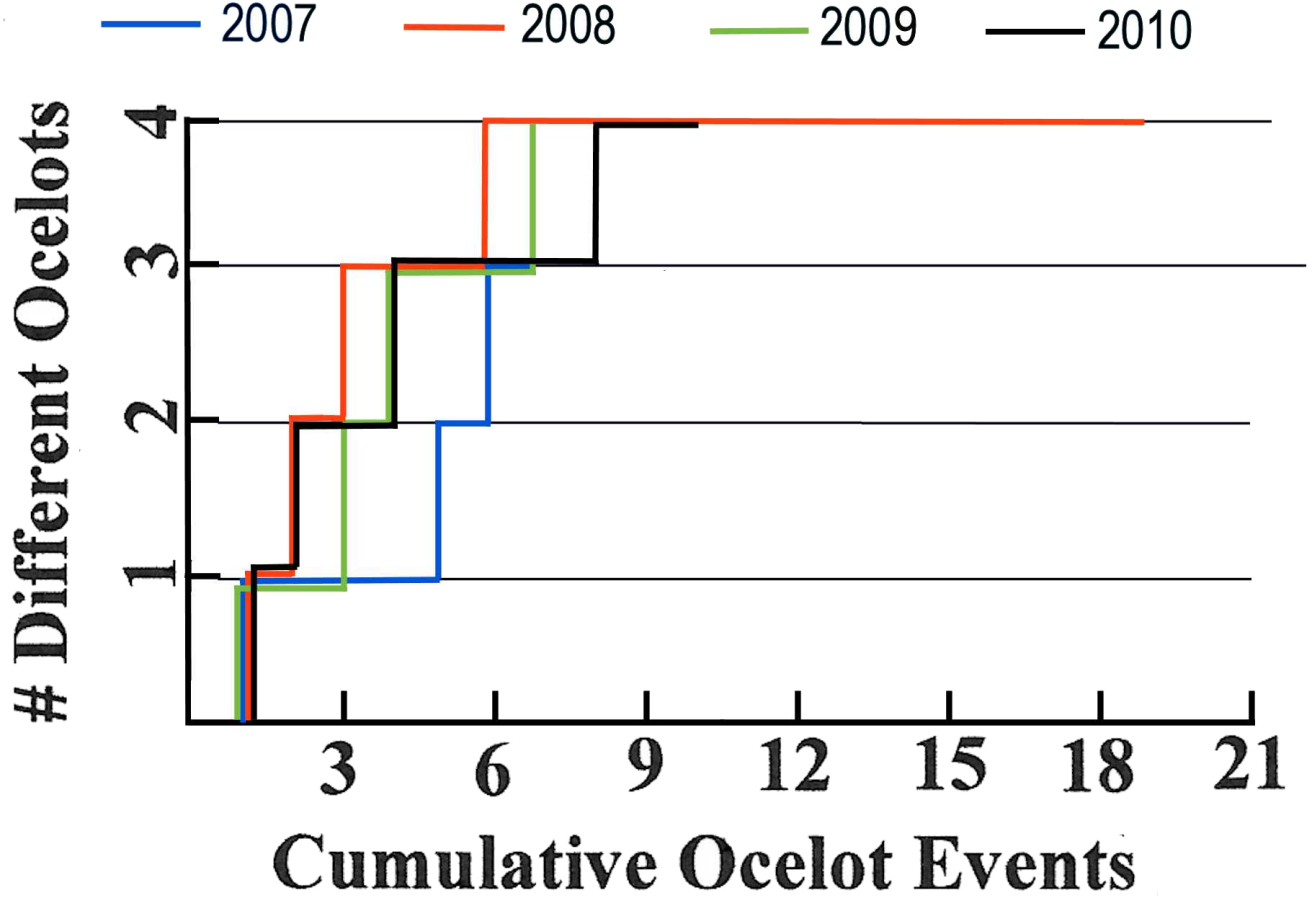
Ocelot accumulation charts for 2007–2010.

**Figure 7:**
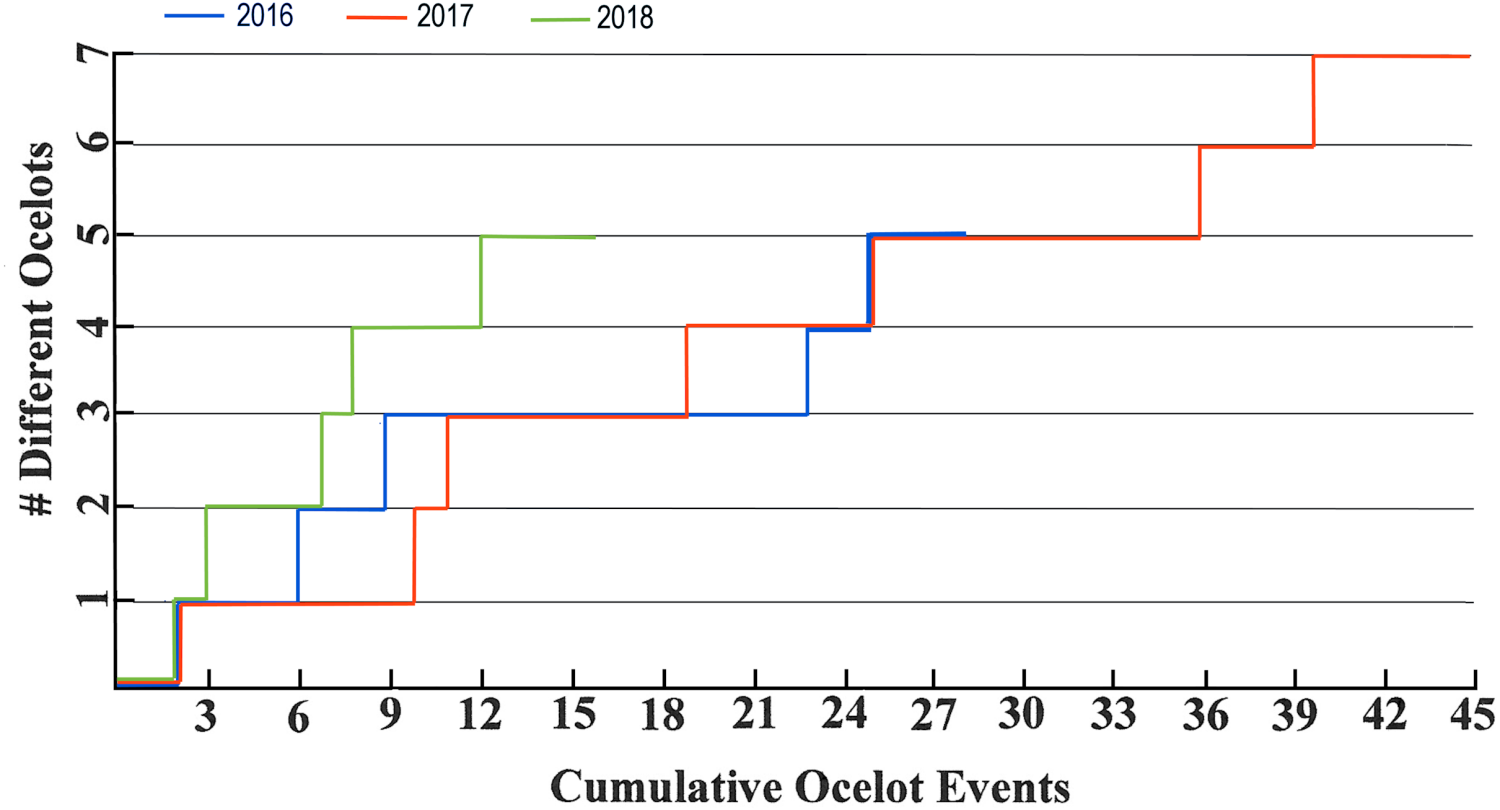
Ocelot accumulation charts for 2016–2018.

A total of at least nine individual ocelots was photographed in the roughly 40 km^2^ study area from 2015–2018 (none were photographed in 2014). At least nine were photographed in the Arroyo Las Palomas area, a subset of the study area (Fig. 2), during 2007–2011 and six were photographed in that area during 2015–2018. Annual numbers of ocelot individuals detected are plotted against cumulative ocelot events for 2007–2010 in Fig. 6 and 2016–2018 in Fig. 7.

**Figure 8:**
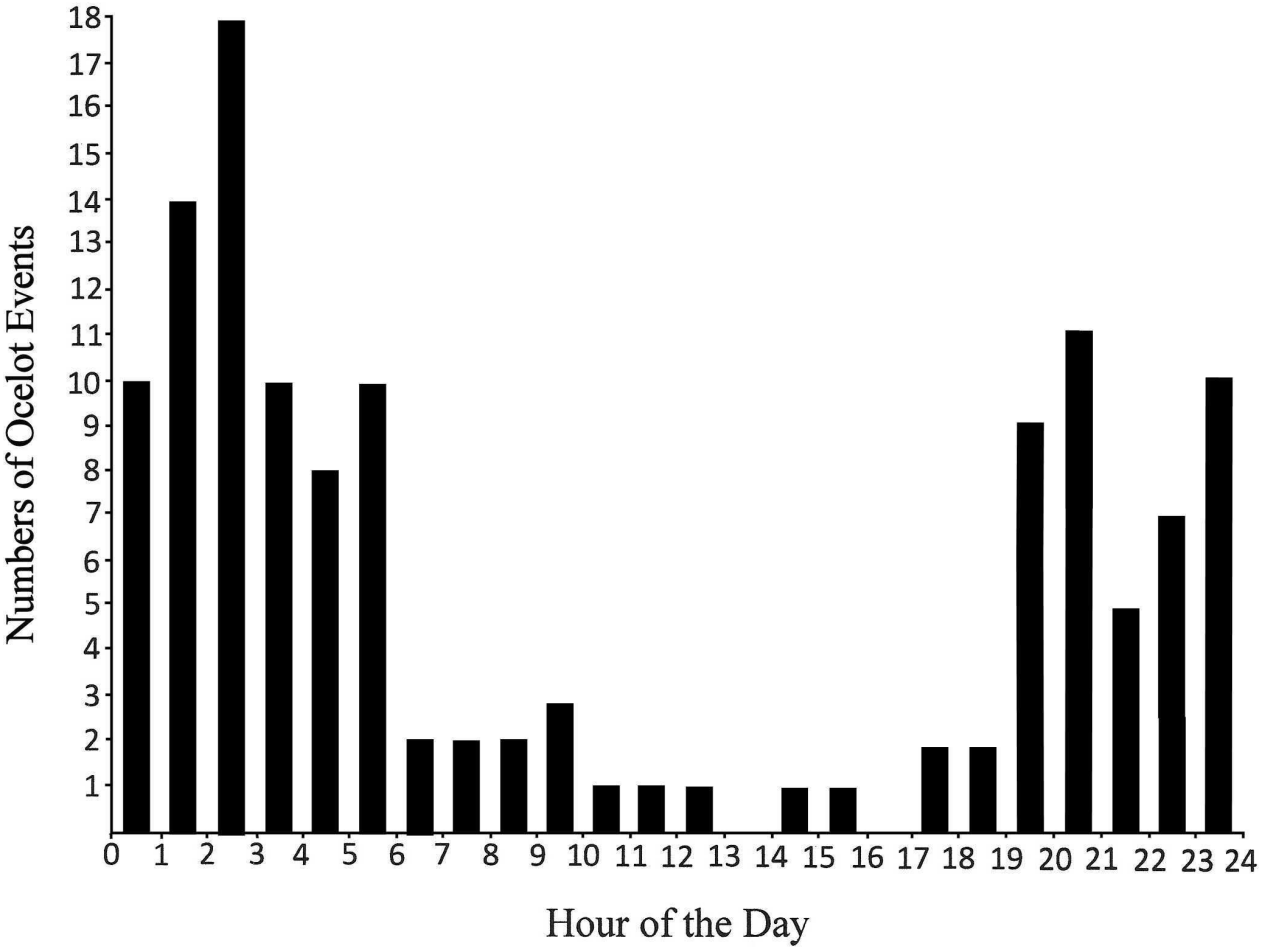
Hourly activity patterns for all ocelots detected during 2007–2018.

Ocelot events are plotted by hour in Fig. 8, which shows that ocelots at Rancho El Aribabi are primarily nocturnal with an activity peak from about 0100 to 0300 hrs. Although mostly nocturnal, we recorded ocelot activity during most hours of the day and night. Ocelots were also photographed every month of the year, although only one event was recorded in June. September and October had the greatest number of events (19 and 16, respectively).

Table 6 details movements by individual ocelots in less than a 24-hour period. These varied from 32–1,221 m (straight-line distance) and movements took from nine minutes to 18.2 h to complete. A male on the Río Cocóspera (R2) moved 1,221 m in 4.2 h. The same ocelot moved 209 m in nine minutes. Although a female and kitten at Arroyo Las Palomas were the only ocelots photographed together, other ocelots were photographed at the same camera site or in close proximity over a relatively short period of time. For instance, on 11 July 2017, LP1 (male) was photographed at T2LP1 at 0035 hrs and LP2 (female) was photographed in the same arroyo (T2LP2), 47 m downstream, at 0155. Two males (LP6, LP7) were photographed at the same site in Arroyo Las Palomas 7–8 January 2008 within four hours and 16 min of each other. These two males were photographed again at another site in the Arroyo Las Palomas area during 16-17 April 2010 within a 27 h period. R1 (female) and R2 (male) were photographed traversing the same log across the Río Cocóspera (site R17) within 24 h of each other on 18–19 September 2017. Two females (LP2, LP4) were photographed at ALP5 within six days of each other in September 2016.

**Table 6:** Short-term (<24 h) movements by individual ocelots.

Ocelot	Moved from^a^/date-time	Moved to^a^/date- time	Distance^b^(m)
R1	R1/24 Mar 17-0312	R17/24 Mar 17-0428	540
R1	R17/15 May 17-0021	R1/15 May 17-0259	540
R1	R1/27 May 17-0200	R17/27 May 17-0231	540
R1	R17/16 Jul 17-0254	R1/16 Jul 17-0351	540
R2	R3/19 Sep 17-1906	R6/20 Sep 17-0119	1,221
R2	R3/22 Nov 17-0557	R15/22 Nov 17-0606	209
LP1	ALP3/26 Nov 16-0117	ALP5/26 Nov 16-0430	378
LP2	T2LP1/11 Jul 17-0903	T2LP2/11 Jul 17-0916	47
LP3	ALP8/6 Nov 17-0240	ALP5/6 Nov 17-2113	535
LP5	ALP8/8 Nov 17-0120	ALP5/8 Nov 17-1933	535
LP5	ALP5/8 Nov 17-1933	ALP6/8 Nov 17-2016	32
LP7	ALP9/3 Apr 08-1918	ALP10/3 Apr 08 -2017	640

**Notes.**

a Code for individual camera sites.

b Straight-line distance.

## Discussion

### Habitat characterization

Ocelots at Rancho El Aribabi were photographed in a mature Fremont cottonwood-Goodding willow gallery forest and associated velvet mesquite bosque along the Río Cocóspera, at an intermittent stream and tributaries thereof in the Sierra Azul amidst an oak-mesquite savanna, and in a Sonoran desertscrub-foothills thornscrub ecotone in the foothills of the Sierra Azul (Fig. 2). They were not photographed at four ridgeline camera sites (919 camera days) or seven cameras at artificial water sources for cattle (2,752 camera days, Table 1). Given that the mean camera days necessary to capture one ocelot was 237 (all cameras) and 125 (at sites where ocelots were photographed), failure to detect ocelots on ridgelines and especially artificial water sources may indicate ocelot avoidance of these types of sites. The upland camera site in a Sonoran desertscrub-foothills thornscrub ecotone that captured two ocelot events in 109 camera days was only 0.14 km from a spring-fed, perennial cattle tank, yet no ocelots were photographed at that tank in 2,268 camera days. Both ridgelines and artificial water sources tend to be quite exposed in our study area, and vegetation within 10–20 m around artificial water sources was often denuded by foraging cattle. Ocelots tend to avoid open areas (Emmons, 1988; Sunquist & Sunquist, 2002; Horne et al., 2009).

Eighteen of 19 camera sites where ocelots were photographed were located in arroyos or along the Río Cocóspera where Fremont cottonwoods, Goodding willows, and/or Arizona sycamore were present in the macro-environment. The one exception, the aforementioned upland camera site, was 0.14 km from a spring-fed cattle tank. Below that cattle tank were a few Goodding willows and a short reach of a perennial or intermittent stream. These three species of riparian trees grow only where there is dependable surface or subsurface water (Felger, Johnson & Wilson, 2001). Mean distance from ocelot camera sites to perennial water was 0.22 km and never exceeded 0.7 km. Mean distance to perennial water from sites where ocelots were not photographed was 0.5 km (Table 3).

The results of the training sample, area under the curve, Hosmer and Lemeshow goodness-of-fit test, and the −2 Log(Likelihood) statistic (Table 3) all indicate the logistic regression model is a reasonably good predictor of ocelot presence. It predicts ocelot absence less precisely, which is to be expected as some of the non-ocelot detection sites were probably suitable for ocelot and if we had extended the camera-trapping effort longer, ocelots may have been detected at those sites. Proximity to perennial water was one of the most important factors in predicting presence of ocelot; however, occurrence of riparian trees in the macro-environment was not, suggesting it may be water that is the key factor and that the riparian trees are simply an indicator of water at or near the surface. Twelve of 22 camera sites along the Río Cocóspera and two in the Arroyo Las Palomas area with riparian trees in the macro-environment failed to produce ocelot images during 2014-2018. We find no information in the literature about ocelot dependence on permanent water, but most places where the species has been studied are mesic, usually tropical areas where water is probably not a limiting factor. Such is not the case in the arid southwestern USA and adjacent Mexico.

The results of the logistic regression and a comparison of numbers of ocelots photographed during periods of cattle presence and absence suggest ocelots in our study area are sensitive to anthropogenic disturbance, including presence of cattle. Distance to paved roads and human habitations were two of the three most important independent variables in the logistic regression equation, and the cattle-dominated anthropogenic influence index was the fourth most important variable. The one paved road in our study area is Mexico Highway 2, which although it is only one lane in each direction, is the primary west-east route in northern Sonora and one of only three paved roads that cross the Sierra Madre Occidental and associated ranges for about 930 km in northwestern Mexico. Highway 2 is very busy with much truck, bus, and passenger car traffic. One of us (SA-V) found a road-killed ocelot on Highway 2 near the entrance to Rancho El Aribabi on 15 March 2013. In 2008, two ocelots were found dead on Highway 15 near Ímuris, about 20 km southwest of La Casona (Avila-Villegas & Lamberton-Moreno, 2013). Roads pose a threat to ocelot populations and connectivity (Haines, Tewes & Laack, 2005; DeYoung & Holbrook, 2010; US Fish and Wildlife Service, 2016). Although apparently avoiding human habitation according to the results of the logistic regression, we photographed male ocelot R2 along the Río Cocóspera only 0.07 km from La Casona.

Periods of cattle use in the Arroyo Las Palomas area were often heavy. Where we had cameras set on tinajas (natural water holes amidst rocks), herds of cattle would often stay on those water holes for hours at a time during the dry season from late April through most of June. They would drink the water down, trample and foul the water, sometimes eliminating surface water during the day, then bed down elsewhere for the night. The images documented water levels rising during the night. These water holes probably dried up sooner in the presence of cattle. So negative effects of cattle on ocelots may be due to effects on water availability or quality, but we cannot rule out other habitat effects or simply the presence of cattle as causative mechanisms.

Dense, low cover is often described as a key feature of ocelot habitat (Sunquist & Sunquist, 2002; Horne et al., 2009). However, the logistic regression did not identify vegetation cover in the two lower strata (≤1.8 m) as particularly important; in fact, mean vegetation cover in these lower strata was lower (23.4%) at ocelot camera sites than at sites where ocelots were not photographed (37.0%, Table 3). It should be noted that we measured vegetation in May, during the dry season. Measurements at these sites during late August or September, toward the end of the rainy season, would have registered much higher cover values in the lower strata as a result of herbaceous vegetation that grows during the summer. So, if wet season vegetation cover is important to ocelots, our analyses would not have detected it.

The results of the logistic regression and other conclusions herein suffer from non-random camera placement design, although camera-trap researchers often elect to place cameras in a grid to obtain representative rather than random samples (Rovero & Spitale, 2016). We acknowledge that our camera locations are probably not proportionally representative of habitats in the study area; riparian sites and oak/mesquite-dominated ephemeral arroyos in the Sierra Azul were oversampled and semi-desert or mesquite grasslands and Sonoran desertscrub-foothills thornscrub ecotone were vastly undersampled. That said, we believe that within the riparian zone of the Río Cocóspera and the arroyos of the Las Palomas area, our camera sites are probably representative of the places used by ocelots and other medium to large mammals. Although our sampling of other types of sites (mesquite grasslands, ridgelines, artificial water sources) was less intensive, we have no reason to believe our results are not broadly representative of those types. Hence, our results and conclusions can be cautiously extrapolated to similar areas within and perhaps outside of our study area.

### Demographics, movements, and other behaviors

MCP is often used as a measure of home range (Mohr, 1947; Guigglio et al., 2006); however, for several reasons our MCPs almost assuredly do not equate to ocelot home range. If we were to consider our calculated 100% MCPs as estimates of home range, they are comparatively small, although the higher estimates (ocelots R1 and R2 in Table 5) are within the range reported in other ocelot studies (see review in US Fish and Wildlife Service, 2016). But ocelot home ranges are almost certainly larger in our study area than the MCPs we calculated. Our MCPs are based on small sample sizes (3–6 localities and 3–25 events), which is likely to result in an underestimate of home range (Guigglio et al., 2006). Our small sample size likely also leads to an underestimate of maximum distance moved. Underestimates of mean maximum distance moved (MMDM), which can be used as a proxy for home range diameter (Karanth & Nichols, 2002; Maffei & Noss, 2008), would in turn underestimate home ranges derived from it. The farthest ocelot movement we documented was only 2.38 km. This contrasts with MMDMs of 3.58 to 10.59 km at the Northern Jaguar Reserve in east-central Sonora (Gómez-Ramírez, Gutiérrez-González & López-González, 2017). Furthermore, ocelots have been known to move 42 km in Arizona (Culver, 2016) and 50 km in Tamaulipas, Mexico (Booth-Binczik, 2007). That said, in our years of camera trapping at Rancho El Aribabi, we documented no movement between the Río Cocóspera and Arroyo Las Palomas areas, which are separated by a minimum of 11.29 km, suggesting movements of that magnitude in our study area likely occur infrequently at best. Another problem for calculating home range (or movements) from our MCPs is that the majority of our ocelots were photographed at the periphery of our study area. Ocelots we detected could have easily been using areas outside of those covered by our camera traps, again resulting in underestimates of home range and distances moved. In addition, the MCP method of determining home range suffers from a number of drawbacks (Worton, 1995; Burgman & Fox, 2003) and does not take into account varying use by an animal within the MCP (Dixon & Chapman, 1980). In summary, although our MCPs are interesting and novel in this northern-most known breeding population of ocelots, they almost certainly underestimate ocelot home ranges and should not be considered a proxy for home range. Their value is that they begin to define ocelot use areas and may help other researchers in future studies better quantify ocelot home ranges at Rancho El Aribabi or elsewhere in the northern extent of the ocelot’s range.

Our study was not designed to determine precise ocelot densities. Sample size, in terms of numbers of ocelot individuals detected, was inadequate for spatial (SCR) models (White et al., 1982), and arrangement of camera traps on the landscape and the long duration of our study meant our data were inappropriate for estimating densities via non-spatial models (Maffei & Noss, 2008; Zimmermann & Foresti, 2016; Gómez-Ramírez, Gutiérrez-González & López-González, 2017). For these reasons, we were not able to calculate precise density estimates.

Figures 6 and 7 show that numbers of ocelots detected yearly during 2007–2010 and 2016–2018 for the most part did not plateau over time, suggesting we had not detected all of the ocelots in the sampled areas. The one exception is 2008, which plateaued at four ocelots and 6-19 events. The only kitten detected was in 2011, hence that cohort is likely under-represented in our data. Probably the best that can be said regarding density, and in the simplest terms, is that at least nine different ocelots used our roughly 4,000 ha study area from 2015 to 2018, and that the distribution of ocelots appeared to be patchy. Nine or more different ocelots used the Arroyo Las Palomas area from 2007 to 2011, and six or more used that area from 2015 to 2018.

Our findings that ocelots were generally solitary and mostly nocturnal are consistent with other studies (Sunquist & Sunquist, 2002). A dearth of ocelot detections in June may be biologically significant. June is typically the hottest and driest month of the year. In the Arroyo Las Palomas area, ocelots may have been limiting their activities to areas near permanent water that were not covered by our cameras. That explanation, however, does not explain why only one ocelot was photographed in June at the Río Cocóspera camera sites, where water is not limiting. We never photographed ocelots engaged in predation or carrying prey. Sunquist & Sunquist (2002) report that ocelots eat most prey where it is captured. Ocelots are reportedly not deterred by water and are strong swimmers (Guggisberg, 1975). Although we obtained many pictures of mammals in water or crossing the Río Cocóspera (e.g., white-tailed deer, collared peccary, coyote), we have no pictures of ocelots in water, but rather we documented two different individuals crossing the Río Cocóspera via a fallen log (18 events). So, in our study area, we have no evidence that ocelots are undeterred by water, but they will use fallen logs to cross a river.

The clustering of cameras on the Río Cocóspera and in the Arroyo Las Palomas area allowed for analysis of short-distance movements not possible with wider-spaced grids or other camera arrangements (Table 6). For instance, ocelot R2 travelled at 23 m/minute between camera sites R13 and R15 (or with greater speed if he did not travel in a straight line). Males and females were photographed at the same or nearby camera site within a short period of time (80 min to six days), indicating temporal overlap of home ranges in both males and females.

Camera sites ALP5 and ALP6 were separated by only 32 m. ALP5 is at the entrance to a narrows in Arroyo Las Palomas and ALP6 is downstream in the narrows, which at that site is a relatively deep, slot canyon with steep canyon walls (but those walls are likely still traversable by an ocelot). Although 20 ocelot events were recorded at ALP5, only two were recorded at ALP6, and only one of those was an ocelot also photographed at ALP5 in the same 24-hour period; in that case, it was 43 min earlier. All ocelots photographed at ALP5 and ALP6 were either moving up or down the arroyo, suggesting that most ocelots traversing the narrows elected to avoid the narrow canyon bottom. We can only speculate as to why that may be the case, but the narrow, exposed canyon bottom may have been perceived as a predation risk by ocelots.

## Conclusions

We documented a relatively small, breeding population of ocelots at the northern edge of the species’ range, about 59 to 68 km SE of the USA-Mexico border at Nogales, Sonora. Ocelots have persisted in the area from at least 2007 to 2018, and have likely occupied the area since at least the 1960s (López-González, Brown & Gallo-Reynoso, 2003). We examined several aspects of the ecology of this population. The only other similar study in northwestern Mexico focused on ocelot survival, abundance, and density at the Northern Jaguar Reserve, approximately 215 km southeast of our study area (Gómez-Ramírez, Gutiérrez-González & López-González, 2017). We employed a non-random camera placement design to maximize the number of ocelot images, but that was accompanied by a loss of robust scientific inference. On the other hand, our study was relatively long, spanning eight years with a four and a half year hiatus between sampling periods. That aspect of our study allowed us to take a longer view of ocelot ecology that is not possible in typical short-term camera trap studies. Although density estimations are best accomplished with short-term studies, estimates of home range and maximum distances moved are better assessed with long-term studies. Non-random clustering of camera sites also allowed examination of short-term ocelot movements not possible with more widely-spaced camera traps.

To our knowledge, no ocelots have been detected in Mexico north of our study area to the USA border, but male ocelots have been detected in recent years in the Santa Rita, Patagonia, and Huachuca mountains in Arizona, approximately 90, 55, and 65 km north and northwest, respectively, of Rancho El Aribabi (Culver, 2016). Although we only documented short-range movements, a male ocelot moved from the Huachuca Mountains to the Patagonia Mountains and back again (42 km one way, Culver, 2016), suggesting ocelots could move from our study area north into the USA. Terrain and vegetation communities similar to those at Rancho El Aribabi lie between our study area and Arizona mountain ranges where ocelots have been documented. To the southeast of Rancho El Aribabi, vegetation communities in the valleys (e.g., Río San Miguel and Río Sonora) quickly turn to subtropical foothills thornscrub with oak and pine-oak woodlands in the mountains. These communities provide connectivity to the southeast and the Northern Jaguar Reserve and other areas where ocelots have been documented (López-González, Brown & Gallo-Reynoso, 2003; Gómez-Ramírez, Gutiérrez-González & López-González, 2017). So although we cannot say how well the ocelot population at Rancho El Aribabi is demographically connected to other such populations, there is a continuity of hospitable landscape that could facilitate movements among distant populations.

Culver (2016) focused camera-trapping efforts in the mountains of southeastern Arizona. Our finding of male and female ocelots in the riparian corridor of the Río Cocóspera suggests that ocelots should be looked for and may have been overlooked in the riparian woodlands of the Santa Cruz and San Pedro rivers in southeastern Arizona. Ocelots in our study area were negatively associated with paved roads, human habitations, and cattle, and proximity to perennial water was important for predicting ocelot presence. These factors should be considered in any strategy to conserve ocelots in northwestern Mexico or Arizona. Planning is underway to reroute Mexico Highway 2 through Rancho El Aribabi, between the Río Cocóspera and Arroyo Las Palomas. Although wildlife undercrossings and other mitigation will likely be part of the new highway design, based on our work, the new road corridor may further isolate ocelots into these two areas of Rancho El Aribabi and may continue to impede north-south movements of ocelots in this binational region. At present, the USA Executive Branch and Congress are debating the construction of additional pedestrian fencing on the USA/Mexico border, and upgrades to existing fences and barriers have been proposed or are under construction on the Arizona-Sonora border. New fencing would likely be exempted from all environmental laws and considerations due to authorizations provided to the USA Department of Homeland Security under the Real ID Act of 2005. The ocelot is one of many species that would be adversely affected by additional fence construction (Peters et al., 2018), and the fence and associated lights, roads, vehicles, and human presence have the potential to sever currently viable ocelot movement corridors across the border, perhaps precluding natural recolonization of suitable habitats in the USA.

##  Supplemental Information

10.7717/peerj.8414/supp-1 Table S1A Ocelots 2014–2018

10.7717/peerj.8414/supp-2 Table S1B Ocelots 2007–2011

10.7717/peerj.8414/supp-3 Table S2 Camera sites and habitat variables

10.7717/peerj.8414/supp-4 Table S3 Camera sites at Rancho El Aribabi

## Additional Information and Declarations

### Field Study Permissions

The following information was supplied relating to field study approvals (i.e., approving body and any reference numbers): Carlos Robles Elias, owner of Rancho El Aribabi, provided verbal permission to access the field sites.

### Data Availability

The following information was supplied regarding data availability: Representative images with locality information, dates, and other data are available for camera sites where ocelots were detected in Greater Good’s Madrean Discovery Expedition database: http://www.madreandiscovery.org/. In the Fauna Database, search for Leopardus pardalis, Mexico, Sonora, County=Imuris.
