# Supplementary material for: Ecology of an ocelot population at the northern edge of the species’ distribution in northern Sonora, Mexico
Source: PeerJ. 2020 Jan 20;8:e8414. doi: 10.7717/peerj.8414 (PMC6977465; doi:10.7717/peerj.8414)
Supplement: Table S1A [file peerj-08-8414-s001.docx]

Supplemental Table 1a. Ocelots 2015-2018

| Event | Date | Time | Location | Ocelot ID | Male/Female |
| --- | --- | --- | --- | --- | --- |
| 1 | 9 Jan 18 | 1920 | T2LP1 | Unidentifiable | ? |
| 2 | 11 Jul 17 | 0035 | T2LP1 | LP1 | Male |
| 3 | 25 Feb 2016 | 0903 | T2LP1 | LP2 | Female |
| 4 | 6 Nov 17 | 0240 | ALP8 | LP3 | ? |
| 5 | 8 Nov 17 | 0120 | ALP8 | LP4 | Female |
| 6 | 17 Mar 18 | 0241 | U1 | U1 | ? |
| 7 | 20 Apr 18 | 2311 | U1 | Unidentifiable | ? |
| 8 | 9 Jan 18 | 2058 | T2LP2 | LP5 | Female |
| 9 | 25 May 17 | 0059 | T2LP2 | LP2 | Female |
| 10 | 11 Jul 17 | 0155 | T2LP2 | LP2 | Female |
| 11 | 12 Oct 17 | 2118 | T2LP2 | LP5 | Female |
| 12 | 23 Sep 17 | 0225 | T2LP2 | LP3 | ? |
| 13 | 13 Feb 2016 | 0507 | T2LP2 | LP2 | Female |
| 14 | 25 Feb 2016 | 0916 | T2LP2 | LP2 | Female |
| 15 | 30 Apr 18 | 0304 | ALP1 | Unidentifiable | ? |
| 16 | 18 Sep 2016 | 0232 | ALP1 | Unidentifiable | ? |
| 17 | 22 Oct 2016 | 0322 | ALP1 | Unidentifiable | ? |
| 18 | 6 Nov 17 | 2113 | ALP5 | LP3 | ? |
| 19 | 8 Nov 17 | 1933 | ALP5 | LP4 | Female |
| 20 | 9 Dec 16 | 0705 | ALP5 | LP3 | ? |
| 21 | 19 Dec 16 | 0156 | ALP5 | LP2 | Female |
| 22 | 26 Nov 16 | 0430 | ALP5 | LP1 | Male |
| 23 | 16 Jul 2016 | 0424 | ALP5 | LP1 | Male |
| 24 | 14 Aug 2016 | 0222 | ALP5 | Unidentifiable | ? |
| 25 | 27 Aug 2016 | 1233 | ALP5 | LP5 | Female |
| 26 | 23 Sep 2016 | 2330 | ALP5 | Unidentifiable | ? |
| 27 | 24 Sep 2016 | 0836 | ALP5 | LP5 | Female |
| 28 | 27 Sep 2016 | 2012 | ALP5 | Unidentifiable | ? |
| 29 | 30 Sep 2016 | 0521 | ALP5 | Unidentifiable | Female |
| 30 | 21 Oct 2016 | 0531 | ALP5 | LP1 | Male |
| 31 | 22 Oct 2016 | 0225 | ALP5 | Unidentifiable | ? |
| 32 | 28 Oct 2016 | 0226 | ALP5 | LP1 | Male |
| 33 | 2 Jul 2018 | 0318 | ALP5 | Unidentifiable | ? |
| 34 | 13 Sep 2018 | 0220 | ALP5 | Unidentifiable | ? |
| 35 | 7 Oct 2018 | 0503 | ALP5 | Unidentifiable | ? |
| 36 | 28 Oct 2018 | 0157 | ALP5 | Unidentifiable | ? |
| 37 | 3 Dec 2018 | 0158 | ALP5 | Unidentifiable | ? |
| 38 | 8 Nov 17 | 2016 | ALP6 | LP4 | Female |
| 39 | 14 Jul 2016 | 0438 | ALP6 | LP2 | Female |
| 40 | 22 Nov 17 | 0606 | R15 | R2 | Male |
| 41 | 22 Nov 17 | 0557 | R3 | R2 | Male |
| 42 | 1 Sep 17 | 0525 | R3 | R2 | Male |
| 43 | 23 Aug 18 | 0053 | R3 | R2 | Male |
| 44 | 18 Sep 17 | 1942 | R3 | R1 | Female |
| 45 | 19 Sep 17 | 1906 | R3 | R2 | Male |
| 46 | 10 Sep 2018 | 0523 | R3 | R1 | Female |
| 47 | 10 Sep 2018 | 0702 | R3 | Unidentifiable |  |
| 48 | 12 Sep 2018 | 0847-0848 | R3 | R3 | Male |
| 49 | 17 Oct 2018 | 1053 | R3 | Unidentifiable | ? |
| 50 | 26 Oct 18 | 2057 | R3 | Unidentifiable | ? |
| 51 | 8 Dec 16 | 0359 | R17 | R1 | Female |
| 52 | 8 Dec 16 | 2234 | R17 | R1 | Female |
| 53 | 12 Dec 16 | 0506 | R17 | R1 | Female |
| 54 | 24 Dec 16 | 0323 | R17 | Unidentifiable | ? |
| 55 | 5 Jan 17 | 1259 | R17 | R1 | ? |
| 56 | 5 Jan 17 | 1823-1832 | R17 | R1 | Female |
| 57 | 24 Jan 17 | 0944 | R17 | R1 | Female |
| 58 | 25 Jan 17 | 1823 | R17 | R1 | Female |
| 59 | 19 Feb 17 | 1913 | R17 | R1 | Female |
| 60 | 19 Feb 17 | 2009 | R17 | R1 | Female |
| 61 | 15 Mar 17 | 2004 | R17 | R1 | Female |
| 62 | 18 Mar 17 | 0036 | R17 | R2 | Male |
| 63 | 24 Mar 17 | 0428 | R17 | R1 | Female |
| 64 | 19 Apr 17 | 0244 | R17 | R1 | Female |
| 65 | 15 May 17 | 0021 | R17 | R1 | Female |
| 66 | 27 May 17 | 0231 | R17 | R1 | Female |
| 67 | 1 Jul 17 | 0250 | R17 | R1 | Female |
| 68 | 16 Jul 17 | 0254 | R17 | R1 | Female |
| 69 | 4 Aug 17 | 0141 | R17 | R2 | Male |
| 70 | 27 Nov 17 | 0050 | R1 | R1 | Female |
| 71 | 24 Mar 17 | 0312 | R1 | R1 | Female |
| 72 | 30 Mar 17 | 2228 | R1 | R1 | Female |
| 73 | 15 May 17 | 0259 | R1 | R1 | Female |
| 74 | 27 May 17 | 0020 | R1 | R1 | Female |
| 75 | 20 Jun 17 | 0247 | R1 | R1 | Female |
| 76 | 16 Jul 17 | 0351 | R1 | R1 | Female |
| 77 | 19 Sep 17 | 0235 | R8 | R1 | Female |
| 78 | 20 Sep 17 | 0119 | R6 | R2 | Male |
| 79 | 1 Sep 17 | 0146 | R5 | Unidentifiable | ? |
| 80 | 16 Aug 17 | 2129 | ALP4 | LP1 | Male |
| 81 | 4 Jan 17 | 2303 | ALP4 | Unidentifiable | ? |
| 82 | 19 Mar 17 | 2241 | ALP4 | LP5 | Female |
| 83 | 24 May 17 | 2322 | R13 | R2 | Male |
| 84 | 26 Nov 16 | 0117 | ALP2 | Unidentifiable | ? |
| 85 | 14 Aug 2016 | 0104 | ALP2 | Unidentifiable | ? |
| 86 | 7 Feb 2016 | 0636 | ALP2 | Unidentifiable | ? |
| 87 | 12 Nov 2015 | 1542 | ALP2 | LP1 | Male |
| 88 | 12 Dec 2015 | 1909 | ALP2 | Unidentifiable | ? |
| 89 | 5 Jan 17 | 2030 | R7 | Unidentifiable | ? |
| 90 | 20 Oct 2016 | 0037-0038 | R14 | Unidentifiable | ? |
| 91 | 22 Oct 2016 | 0003 | R14 | Unidentifiable | ? |
