# Supplementary material for: Ecology of an ocelot population at the northern edge of the species’ distribution in northern Sonora, Mexico
Source: PeerJ. 2020 Jan 20;8:e8414. doi: 10.7717/peerj.8414 (PMC6977465; doi:10.7717/peerj.8414)
Supplement: Table S1B [file peerj-08-8414-s002.docx]

1. Supplemental Table 1b. Ocelots 2007-2011

| Event | Date | Time | Location | Ocelot ID | Male/Female |
| --- | --- | --- | --- | --- | --- |
| 1 | 1-Jul-07 | 4:53 | ALP11 | LP6 | M |
| 2 | 5-Jul-07 | 8:25 | ALP11 | LP6 | M |
| 3 | 5-Jul-07 | 1:38 | ALP11 | LP6 | M |
| 4 | 28-Aug-07 | 6:47 | ALP11 | LP6 | M |
| 5 | 28-Aug-07 | 3:02 | ALP11 | LP9 | ? |
| 6 | 6-Nov-07 | 2:27 | ALP10 | LP10 | ? |
| 7 | 28-Dec-07 | 19:57 | ALP11 | LP6 | M |
| 8 | 7-Jan-08 | 20:56 | ALP11 | LP7 | M |
| 9 | 8-Jan-08 | 1:12 | ALP11 | LP6 | M |
| 10 | 8-Jan-08 | 13:30 | ALP9 | LP11 | M |
| 11 | 8-Jan-08 | 13:47 | ALP9 | LP11 | M |
| 12 | 9-Feb-08 | 2:28 | ALP11 | LP7 | M |
| 13 | 13-Feb-08 | 23:04 | ALP10 | LP8 | F |
| 14 | 13-Feb-08 | 23:06 | ALP10 | LP8 | F |
| 15 | 16-Feb-08 | 23:02 | ALP11 | Unidentifiable | ? |
| 16 | 21-Feb-08 | 20:43 | ALP9 | LP7 | M |
| 17 | 6-Mar-08 | 5:47 | ALP9 | LP7 | M |
| 18 | 9-Mar-08 | 5:17 | ALP10 | LP6 | M |
| 19 | 3-Apr-08 | 19:18 | ALP9 | LP7 | M |
| 20 | 3-Apr-08 | 20:17 | ALP10 | LP7 | M |
| 21 | 4-Apr-08 | 4:18 | ALP9 | LP8 | F |
| 22 | 15-Apr-08 | 23:04 | T2LP5 | LP7 | M |
| 23 | 7-Oct-08 | 4:03 | ALP11 | LP7 | M |
| 24 | 27-Oct-08 | 2:16 | ALP11 | LP7 | M |
| 25 | 20-Dec-08 | 22:17 | ALP9 | LP7 | M |
| 26 | 20-Dec-08 | 23:34 | ALP11 | LP6 | M |
| 27 | 5-Jan-09 | 21:06 | ALP9 | LP7 | M |
| 28 | 31-May-09 | 22:24 | ALP11 | LP7 | M |
| 29 | 26-Aug-09 | 23:19 | ALP11 | LP8 | F |
| 30 | 16-Sep-09 | 3:49 | ALP11 | LP6 | M |
| 31 | 29-Sep-09 | 4:00 | ALP9 | LP6 | M |
| 32 | 2-Oct-09 | 22:23 | ALP11 | LP8 | F |
| 33 | 30-Oct-09 | 1:07 | ALP11 | LP12 | M |
| 34 | 2-Apr-10 | 19:06 | ALP9 | LP7 | M |
| 35 | 10-Apr-10 | 3:35 | ALP11 | LP8 | F |
| 36 | 16-Apr-10 | 21:00 | ALP9 | LP7 | M |
| 37 | 17-Apr-10 | 23:48 | ALP9 | LP6 | M |
| 38 | 10-May-10 | 3:35 | ALP9 | LP7 | M |
| 39 | 22-Jul-10 | 1:34 | ALP11 | Unidentifiable | ? |
| 40 | 23-Nov-10 | 23:17 | ALP11 | LP6 | M |
| 41 | 3-Dec-10 | 15:37 | T2LP6 | LP13 | M |
| 42 | 13-Dec-10 | 20:48 | T2LP6 | LP13 | M |
| 43 | 13-Dec-10 | 22:47 | ALP11 | LP7 | M |
| 44 | 21-Jan-11 | 21:37 | ALP11 | LP7 | M |
| 45 | 12-Feb-11 | 11:38 | ALP11 | LP8, LP14 | F, ? (kitten) |
