# Supplementary material for: Ecology of an ocelot population at the northern edge of the species’ distribution in northern Sonora, Mexico
Source: PeerJ. 2020 Jan 20;8:e8414. doi: 10.7717/peerj.8414 (PMC6977465; doi:10.7717/peerj.8414)
Supplement: Table S2 [file peerj-08-8414-s003.docx]

Supplemental Table 2. 2014-2018 Rancho El Aribabi camera sites and habitat variables used in binary logistic regression.

| Camera Site | Ocelot, 1=yes  0=no | distance to permanent water (km) | distance to paved road (km) | distance to unpaved road (km) | distance to human habitation (km) | presence (1) or absence (0) of cottonwood, willow, or sycamore (macro vegetation community) | % canopy cover (>1.8 m) | % cover in the shrub and ground layer (< 1.8 m) | anthropogenic influences index |
| --- | --- | --- | --- | --- | --- | --- | --- | --- | --- |
| R1 | 1 | 0.04 | 2.77 | 0.53 | 1.22 | 1 | 200 | 3.0 | 1.30 |
| R2 | 0 | 0 | 2.53 | 0.64 | 1.02 | 1 | 117 | 72 | 0.93 |
| R3 | 1 | 0.08 | 2.53 | 0.69 | 1.11 | 1 | 40 | 11 | 0.16 |
| R4 | 0 | 0.07 | 2.45 | 0.57 | 1.00 | 1 | 127 | 39 | 3.36 |
| R5 | 1 | 0.02 | 2.00 | 0.39 | 0.49 | 1 | 71 | 2.1 | 6.29 |
| R6 | 1 | 0 | 1.45 | 0.04 | 0.07 | 1 | 108 | 77 | 0.90 |
| R7 | 1 | 0.03 | 2.00 | 0.44 | 0.52 | 1 | 67 | 11 | 0.12 |
| R8 | 1 | 0.003 | 1.88 | 0.13 | 0.25 | 1 | 150 | 9.5 | 1.05 |
| R9 | 0 | 0.003 | 1.51 | 0.18 | 0.18 | 1 | 73 | 102 | 8.76 |
| R10 | 0 | 0.1 | 1.24 | 0.02 | 0.31 | 1 | 155 | 31 | 0.84 |
| R11 | 0 | 0.03 | 1.91 | 0.09 | 0.36 | 1 | 140 | 73 | 8.54 |
| R12 | 0 | 0 | 1.38 | 0.02 | 0.13 | 1 | 102 | 133 | 7.18 |
| R13 | 1 | 0.07 | 2.35 | 0.58 | 0.88 | 1 | 71 | 40 | 1.14 |
| R14 | 1 | 0.03 | 1.79 | 0.23 | 0.22 | 1 | 41 | 65 | 0.73 |
| R15 | 1 | 0.03 | 2.34 | 0.51 | 0.93 | 1 | 123 | 25 | 0.19 |
| R16 | 0 | 0 | 2.07 | 0.46 | 0.56 | 1 | 118 | 51 | 0 |
| R17 | 1 | 0 | 2.20 | 0.54 | 0.73 | 1 | 139 | 63 | 1.13 |
| R18 | 0 | 0 | 2.42 | 0.57 | 1.00 | 1 | 119 | 19 | 0 |
| R19 | 0 | 0.003 | 1.64 | 0.13 | 0.11 | 1 | 48 | 71 | 1.21 |
| R20 | 0 | 0.18 | 1.18 | 0.01 | 0.38 | 0 | 141 | 11 | 0.74 |
| R21 | 0 | 0.004 | 1.56 | 0 | 0.02 | 1 | 4.5 | 71 | 9.79 |
| R22 | 0 | 0.03 | 2.14 | 0.54 | 0.66 | 1 | 173 | 14 | 0.17 |
| RG1 | 0 | 0.86 | 7.01 | 0 | 6.01 | 0 | 0 | 35 | 7.29 |
| RG2 | 0 | 0.83 | 2.29 | 0 | 0.79 | 0 | 22 | 51 | 3.43 |
| RG3 | 0 | 0.91 | 4.25 | 0 | 1.77 | 0 | 14 | 14 | 0.81 |
| RG4 | 0 | 1.45 | 4.51 | 0 | 2.31 | 0 | 36 | 40 | 1.45 |
| CW1 | 0 | 0 | 3.81 | 0 | 1.10 | 0 | 0 | 2.7 | 1.10 |
| CW2 | 0 | 1.65 | 2.80 | 0.45 | 2.04 | 0 | 10 | 0 | 12.0 |
| CW3 | 0 | 1.53 | 2.82 | 0.38 | 2.00 | 0 | 55 | 51 | 4.0 |
| CW4 | 0 | 0.003 | 7.86 | 0.09 | 7.23 | 1 | 27 | 3.3 | 0.47 |
| CW5 | 0 | 0 | 7.85 | 0.07 | 7.22 | 1 | 91 | 18 | 4.02 |
| CW6 | 0 | 0.01 | 7.85 | 0.07 | 7.22 | 0 | 15 | 90 | 1.66 |
| CW7 | 0 | 2.51 | 5.50 | 0 | 5.00 | 0 | 0 | 17 | 375 |
| U1 | 1 | 0.14 | 7.86 | 0.20 | 7.16 | 0 | 9.7 | 42 | 0 |
| U2 | 0 | 0.42 | 1.96 | 0.19 | 0.36 | 0 | 57 | 33 | 2.51 |
| U3 | 0 | 1.43 | 2.83 | 0.20 | 1.88 | 0 | 22 | 56 | 36.2 |
| U4 | 0 | 1.25 | 2.70 | 0.02 | 1.59 | 0 | 11 | 14 | 31.6 |
| U5 | 0 | 0.09 | 1.64 | 0.02 | 0.03 | 0 | 37 | 35 | 2.25 |
| T2LP1 | 1 | 0.58 | 10.94 | 0.02 | 9.70 | 1 | 94 | 22 | 10.91 |
| T2LP2 | 1 | 0.53 | 10.94 | 0.02 | 9.69 | 1 | 120 | 29 | 0.76 |
| T2LP3 | 0 | 0.68 | 10.13 | 0.02 | 8.99 | 1 | 40 | 7.6 | 0.07 |
| T2LP4 | 0 | 1.11 | 9.68 | 0 | 8.59 | 1 | 58 | 34 | 3.95 |
| ALP1 | 1 | 0.62 | 11.31 | 0.01 | 10.06 | 1 | 83 | 6.4 | 3.67 |
| ALP2 | 1 | 0.70 | 11.16 | 0.17 | 9.89 | 1 | 70 | 0 | 2.10 |
| ALP3 | 0 | 0.70 | 11.16 | 0.17 | 9.89 | 1 | 83 | 4.3 | 7.61 |
| ALP4 | 1 | 0.55 | 10.97 | 0.29 | 9.79 | 1 | 74 | 6.0 | 0.66 |
| ALP5 | 1 | 0.36 | 10.75 | 0.49 | 9.60 | 1 | 23 | 0 | 7.54 |
| ALP6 | 1 | 0.31 | 10.74 | 0.50 | 9.59 | 1 | 0 | 28 | 1.69 |
| ALP7 | 0 | 0.46 | 10.89 | 0.40 | 9.69 | 1 | 85 | 6.0 | 3.76 |
| ALP8 | 1 | 0 | 11.95 | 0.63 | 10.74 | 1 | 19 | 4.2 | 43.36 |
