# Supplementary material for: Ecology of an ocelot population at the northern edge of the species’ distribution in northern Sonora, Mexico
Source: PeerJ. 2020 Jan 20;8:e8414. doi: 10.7717/peerj.8414 (PMC6977465; doi:10.7717/peerj.8414)
Supplement: Table S3 [file peerj-08-8414-s004.docx]

3) Camera Sites, Rancho El Aribabi

| 2014-2018 Camera Sites | | | | | | | |
| --- | --- | --- | --- | --- | --- | --- | --- |
| Camera # | Type | Coordinates/  Elevation | Vegetation | Dates | Camera Days | # images | Ocelot? |
| R1 | River | 30.84819, -110.67495, 989 m | Riverine riparian | 20 Nov 16 to 30 May 18 | 483 | 4119 | Y |
| R2 | River log crossing | 30.850767, -110.674916, 982 m | Riverine riparian | 9 Feb to 30 May 18 | 111 | 643 | N |
| R3 | Tributary to River | 30.850722 -110.675606, 995 m | Mesquites, hackberry | 14 May 16 to 6 Nov 18 | 866 | 41348, 317v, | Y |
| R4 | River | 30.849190, -110.674104, 982 m | Riverine riparian | 7 Mar 15 to 28 May 18 | 1133 | 9446 | N |
| R5 | River | 30.852976, -110.667872, 992 m | Riverine riparian | 12 May 17 to 30 May 18 | 387 | 6496 | Y |
| R6 | River | 30.856531, -110.664984, 984 m | Riverine riparian | 15 Aug 16 to 30 May 18 | 564 | 12891 | Y |
| R7 | Mouth of tributary to the river | 30.844975 -110.681096, 985 m | Mesquites | 5 Jul 14 to 30 May 18 | 947 | 21632 | Y |
| R8 | Mouth of tributary to the river | 30.855531, -110.666206, 972 m | Riverine riparian | 7 Mar 15 to 6 Sep 18 | 1115 | 6793 | Y |
| R9 | River | 30.856145, -110.664631, 983 m | Riverine riparian | 23 Aug 15 to 28 May 18 | 804 | 24347 | N |
| R10 | Mouth of tributary to the river | 30.857324, 110.665707, 993 m | Mesquite-hackberry thicket | 7 Mar 15 to 9 Aug 17 | 359 | 1125 | N |
| R11 | River | 30.853284, -110.668164, 996 m | Mesquite thicket | 10 Feb 17 to 18 Aug 17 | 151 | 1716 | N |
| R12 | River | 30.857808, -110.665192, 983 m | Cienega with willows | 10 Feb to 12 May 17 | 92 | 1822 | N |
| R13 | River | 30.852114, -110.672520, 982 m | Mesquites and cottonwoods | 12-25 May 17 | 14 | 9132 | Y |
| R14 | River | 30.853657 -110.666102, 990 m | Mesquite bosque | 3 Sep 16 to 12 Nov 16 | 71 | 621 | Y |
| R15 | Mouth of tributary to the river | 30.852273, -110.674680, 981 m | Riverine riparian | 11 Apr 15 to 28 May 18 | 670 | 3291 | Y |
| R16 | Near mouth of Cañon Puma, River | 30.852836, -110.666568, 992 m | Riverine riparian | 3-7 July 17 | 5 | 3662 | N |
| R17 | Log crossing of the river | 30.85248, -110.67233, 968 m | Riverine riparian | 19 Nov 16 to 19 Aug 17 | 274 | 3427 | Y |
| R18 | River, log crossing of the river | 30.851333, -110.675140 984 m | Riverine riparian | 20 Nov 16 to 19 Aug 17 | 273 | 3724 | N |
| R19 | River | 30.855031, -110.663971, 982 m | Riverine riparian | 7 Mar 15 to 15 Aug 16 | 335 | 825 | N |
| R20 | Mouth of tributary to the river | 30.859832, -110.665906, 995 m | Mesquite thicket | 28 Nov 15 to 9 Feb 18 | 432 | 1705 | N |
| R21 | River, Road on W bank | 30.856109, -110.665457, 986 m | Disturbed area adjacent to riverine riparian | 3 Oct 15 to 28 Nov 15 | 56 | 650 | N |
| R22 | River | 30.85227, -110.67153, 984 m | Mesquite thicket | 10 July 15 to 25 Sep 15 | 78 | 31 | N |
| RG1 | Ridgeline road | 30.834421, -110.593623, 1305 m | Mesquite grassland | 19 Nov 16 to 12 Apr 18 | 390 | 11833 | N |
| RG2 | Ridgeline road | 30.84763, -110.65978, 1097 m | Mesquite grassland | 19 Nov 16 to 19 Aug 17 | 274 | 6554 | N |
| RG3 | Just off a ridgeline road | 30.83132, -110.64577, 1149 m | Mesquite grassland | 19 Nov 16 to 28 Mar 17 | 130 | 11344 | N |
| RG4 | Just off a ridgeline road | 30.83064, -110.64014, 1169 m | Mesquite grassland | 19 Nov 16 to 23 Mar 17 | 125 | 12409 | N |
| CW1 | Cattle water | 30.83398, -110.65468, 1098 | Mesquite grassland | 19 Nov 16 to 20 Feb 17 | 94 | 11679 | N |
| CW2 | Cattle Tank | 30.845466, 110.644827, 1039 m | Mesquite grassland | 9 Sep 14 to 2 Oct 15 | 76 | 11127 | N |
| CW3 | Cattle Tank | 30.845174, -110.645819, 1038 m | Mesquite bosque | 10 Mar 15 to 11 May 18 | 304 | 14491 | N |
| CW4 | Cattle Tank | 30.827976, -110.588042, 1312 m | Mesquite-oak woodland | 5 Jul 14 to 7 Jul 16,10 Feb 18 to 29 May 18 | 462 | 17036 | N |
| CW5 | Cattle Tank | 30.827962, -110.588534, 1310 m | Mesquite-oak woodland | 5 Jul 14 to 2 Sep 16, 12 May 17 to 29 May 18 | 1172 | 16253 | N |
| CW6 | Cattle Tank | 30.82789, -110.58842, 1323 m | Mesquite-oak woodland | 2 Sep 16 to 12 May 17 | 634 | 3459 | N |
| CW7 | Cattle trough | 30.837097, -110.612386, 1124 m | Mesquite grassland | 19 to 28 Aug 17 | 10 | 9804 | N |
| U1 | Uplands | 30.827025, -110.589412. 1300 m | Sonoran desertscrub/foothills thornscrub | 10 Feb 18 to 29 May 2018 | 109 | 3330 | Y |
| U2 | Mesquite slope | 30.85118, -110.66171, 1037 m | Mesquite bosque | 19 Nov 16 to 19 Aug 17 | 274 | 4092 | N |
| U3 | Uplands | 30.844865, -110.646572, 1025 m | Mesquite grassland | 3 Oct 15 to 10 Jul 16 | 30 | 5127 | N |
| U4 | Arroyo Seco | 30.844696, -110.650693, 1023 m | Mesquite-hackberry lined arroyo in mesquite grassland | 3 Sep 16 to 22 May 18 | 523 | 6726 | N |
| U5 | Trash pile | 30.855723, -110.665995, 996 m | Mesquite grassland | 13-14 May 16, 7-9 Dec 18 | 4 | 174 | N |
| T2LP1 | Ephemeral stream | 30.81917, -110.55399, 1347 m | Oak savanna with scattered cottonwoods in the arroyo | 5 Jul 14 to 23 Jan 18 | 507 | 10235 | Y |
| T2LP2 | Ephemeral stream | 30.81997, -110.55452, 1345 m | Oak savanna with scattered cottonwoods in the arroyo | 7 Mar 15 to 29 May 18 | 847 | 8482 | Y |
| T2LP3 | Ephemeral stream | 30.822365, 110.561101, 1358 m | Oak savanna with scattered cottonwoods in the arroyo | 7 Mar 15 to 22 Aug 15 | 43 | 2135 | N |
| T2LP4 | Ephemeral stream | 30.824263, -110.566287, 1406 m | Arroyo with scattered cottonwoods in mesquite-oak savanna | 28 Nov 15 to 4 Nov 2017 | 712 | 3423 | N |
| ALP1 | Ephemeral stream | 30.819620, -110.548616, 1345 m | Scattered cottonwoods & sycamores in an oak savanna | 3 Oct 15 to 29 May 18 | 876 | 5360 | Y |
| ALP2 | Ephemeral stream | 30.82061, -110.54879, 1346 m | Scattered cottonwoods & sycamores in an oak savanna | 5 Jul 14 to 23 Feb 18 | 774 | 7916 | Y |
| ALP3 | Ephemeral stream | 30.82061, -110.54879, 1346 m | scattered cottonwoods & sycamores in an oak savanna | 3 Oct 15 to 13 Jun 17 | 672 | 7930 | N |
| ALP4 | Ephemeral stream | 30.821530, -110.549641, 1323 m | scattered cottonwoods & sycamores in an oak savanna | 7 Mar 15 to 22 Apr 18 | 934 | 27306 | Y |
| ALP5 | Ephemeral stream | 30.819620, -110.548616, 1333 m | scattered cottonwoods & sycamores in an oak savanna | 3 Oct 15 to 8 Dec 18 | 1091 | 12057 | Y |
| ALP6 | Ephemeral stream | 30.823499, -110.551544, 1333 m | scattered cottonwoods & sycamores in an oak savanna | 3 Oct 15 to 29 May 18 | 839 | 4319 | Y |
| ALP7 | Ephemeral stream | 30.822229, -110.550164, 1323 m | scattered cottonwoods & sycamores in an oak savanna | 6 Oct 15 to 10 May 17 | 387 | 3872, 716v | N |
| ALP8 | Ephemeral stream | 30.814968, -110.544007, 1382 m | scattered cottonwoods & sycamores in an oak savanna | 13 May 17 to 29 May 18 | 11 | 1067 | Y |
| 2007-2011 Ocelot Camera Sites | | | | | | | |
| Camera # | Type | Coordinates/Elevation | | Vegetation | | | |
| ALP9 | Ephemeral Stream | 30.824855, -110.556183, 1277 m | | scattered cottonwoods & sycamores in an oak savanna | | | |
| ALP10 | Ephemeral Stream | 30.822161, -110.550310, 1321 m | | scattered cottonwoods & sycamores in an oak savanna | | | |
| ALP11 | Ephemeral Stream | 30.807416, -110.541848, 1401 m | | scattered cottonwoods & sycamores in an oak savanna | | | |
| T2LP5 | Ephemeral Stream | 30.820801, -110.554772, 1321 m | | scattered cottonwoods & sycamores in an oak savanna | | | |
| T2LP6 | Ephemeral Stream | 30.829726, -110.562877, 1266 m | | scattered cottonwoods & sycamores in an oak savanna | | | |
